# Laboratory‐Detected *Clostridioides difficile* Stool‐Test Positivity and Mortality in Sepsis: A Retrospective Cohort Study

**DOI:** 10.1002/hsr2.73206

**Published:** 2026-09-07

**Authors:** Jiyun Hu, Fuxing Deng, Tingyue Hu, Binbin Meng, Songyun Deng, Lina Zhang, Wenchao Li

**Affiliations:** ^1^ Department of Critical Care Medicine, Hunan Provincial Clinical Research Center for Critical Care Medicine, National Clinical Research Center for Geriatric Disorders, Xiangya Hospital Central South University Changsha Hunan PR China; ^2^ Department of Gastroenterology Lixian People's Hospital Lixian Hunan PR China; ^3^ Emergency Department of Internal Medicine Emergency Trauma Center, The First Affiliated Hospital of Xinjiang Medical University Urumqi Xinjiang PR China

**Keywords:** *Clostridioides difficile*, marker, prognosis, sepsis, stool pathogens

## Abstract

**Background:**

Sepsis disrupts intestinal microbiota, and broad‐spectrum antibiotics may worsen dysbiosis. *Clostridioides difficile* (*C. difficile*) infection (CDI) is a prevalent nosocomial enteric pathogen among critically ill patients, yet its prognostic implications in sepsis remain inadequately studied.

**Methods:**

We retrospectively analyzed 13,484 adult sepsis hospitalizations from MIMIC‐IV. The exposure was any laboratory‐detected C. difficile‐positive stool test during the index hospitalization. Cox regression, detailed early‐antibiotic adjustment, stabilized inverse probability weighting, timing‐ and assay‐based sensitivity analyses, and a post hoc nested‐model comparison assessed association and incremental prediction. Archived positive‐subgroup machine‐learning models were retained as exploratory risk‐stratification analyses.

**Results:**

Detailed early‐antibiotic adjustment substantially attenuated the association: the 30‐day hazard ratio (HR) was 0.98 (95% CI, 0.87–1.11; *p* = 0.767) and the 90‐day HR was 1.08 (95% CI, 0.99–1.18; *p* = 0.103). Early, landmark, PCR‐only, and toxin‐positive analyses were also nonsignificant, whereas the stabilized‐weighted 90‐day estimate remained modest (HR, 1.13; 95% CI, 1.03–1.24; *p* = 0.010), indicating inconsistency across analyses. Predictive models showed strong performance (C‐index: 0.81 for in‐hospital mortality; 0.68 for 30‐day mortality).

**Conclusions:**

While laboratory‐detected *C. difficile* stool‐test positivity is associated with worse unadjusted outcomes in sepsis, this relationship is substantially attenuated and inconsistent after rigorous adjustment. Accordingly, laboratory positivity may serve as a marker of a complex clinical phenotype. Evidence for its independent prognostic utility remains limited, and its incremental predictive value beyond conventional severity measures is minimal.

## 1 Introduction

Sepsis is a significant public health issue, resulting in an estimated 11 million deaths worldwide annually [1, 2]. It is a syndrome in which an infection triggers a dysregulated host response, leading to life‐threatening organ dysfunction. A 2020 research report on Intensive Care Units (ICUs) in 44 hospitals in China revealed that the incidence rate of sepsis was 20.6%, with a case fatality rate of 35.5% [3]. The case fatality rate of severe sepsis was found to be 50% or higher, which places a significant financial burden on both patients and medical professionals [4]. To improve sepsis prognosis, it's vital to promptly assess disease severity, control infection sources, manage inflammatory responses, restore organ function, and deliver personalized interventions [5].

There is ongoing debate regarding the optimal timing and selection of antibiotics in sepsis management, with varying recommendations based on factors such as patient population, pathogen profile, and local antibiotic resistance patterns [6, 7, 8]. The “golden hour” of antibiotics is critical for patients with sepsis, where early antibiotic administration within the first hour of recognition of sepsis symptoms is associated with improved outcomes. However, inappropriate antibiotic use not only harms individual patients by fostering antibiotic resistance and increasing the risk of adverse effects like *Clostridium difficile* infection, but also contributes to the global health threat of antimicrobial resistance [9, 10]. Sepsis patients with antibiotics often experience an imbalance in their intestinal microecology, which can be characterized by a reduction in beneficial bacteria, excessive proliferation of harmful bacteria, or increased colonization of pathogenic bacteria [11]. Notably, clindamycin, later‑generation cephalosporins, and fluoroquinolones consistently carry high CDI risk [12]. Antibiotic selection can disrupt intestinal flora, potentially leading to conditions such as *C. difficile* infection or dysbiosis [13]. In critical illness and sepsis, the intestinal ecosystem often shifts toward pathobiont dominance; barrier integrity falters; and microbial translocation contributes to organ dysfunction—mechanistic links that make gut health central to outcomes [14]. *Clostridioides difficile* (formerly *Clostridium difficile*) is the leading healthcare‐associated enteric pathogen; the genus reassignment was formally validated in 2016 and is now standard usage.

Evaluating the intestinal microecological status and potential sources of infection in patients can be achieved through stool bacterial culture, which is an important diagnostic tool. Stool bacterial culture can aid in identifying potential pathogenic bacteria, such as *C. difficile* [15, 16]. These strains may cause endogenous intestinal infections or sepsis by translocating into the bloodstream [17]. Additionally, stool bacterial culture indirectly reflects the status of intestinal barrier function [18, 19]. *Clostridioides difficile* (formerly Clostridium *difficile*) is the leading healthcare‑associated enteric pathogen; the genus reassignment was formally validated in 2016 and is now standard usage [20]. CDI pathogenesis is toxin‑mediated (TcdA/TcdB), but asymptomatic colonization is common in hospitals and long‑term care, complicating interpretation of positive tests. Pooled estimates suggest ~8%–10% colonization among adults at hospital admission, with higher rates in specific settings [21]. CDI is strongly associated with worse hospital outcomes—including longer length of stay, higher costs, and increased mortality—particularly in the critically ill. Meta‑analyses and ICU‑focused reviews document increased in‑hospital mortality and prolonged ICU/hospital stay among patients with CDI [22]. Evidence directly quantifying CDI's prognostic impact in rigorously defined sepsis cohorts remains limited: in a multicenter U.S. cohort of hospitalized sepsis patients, hospital‑onset CDI increased in‑hospital mortality and length of stay, but longer‑term survival and causal‑inference estimates were not assessed.

In this study, the exposure of interest is laboratory‐detected *C. difficile* stool‐test positivity during the index hospitalization. This *C. difficile*‐specific framing is biologically and clinically coherent: (i) antibiotics used in sepsis perturb the microbiome in ways that predispose to *C. difficile* colonization or infection; (ii) *C. difficile*‐associated gut barrier injury can amplify systemic inflammation; and (iii) *C. difficile* has repeatedly been linked with adverse outcomes in ICU populations. This retrospective cohort study aims to evaluate the association between laboratory‐detected *C. difficile* stool‐test positivity and key clinical endpoints, including in‐hospital mortality and 30‐ and 90‐day survival outcomes among critically ill patients with sepsis. Secondary analyses use regression and machine‐learning models to evaluate prognostic performance.

## 2 Method

### 2.1 Data Source

The Medical Information Mart for Intensive Care (MIMIC) IV database comprises clinical data from 299,712 adult patients aged ≥ 16 years [23]. MIMIC‐IV, a collaborative effort between MIT and Harvard Medical School, aggregates data from ICU patients at Harvard University's Beth Israel Deaconess Medical Center spanning from June 2008 to 2019. Access to the MIMIC‐IV dataset is granted to researchers worldwide following a stringent privacy protection process and obtaining joint approval from the ethical review boards of MIT and Harvard Medical School. Researchers are required to complete an online course from the National Institutes of Health and pass an ethics exam before accessing the database (ID: 48389172). Individual patient consent is not required as the project will not affect clinical care and all sensitive health information will remain anonymous. MIMIC‐IV utilizes a modular data organization method, emphasizes data sources, and facilitates the use of different data sources individually or jointly, in comparison to MIMIC‐III.

### 2.2 Study Population

**Figure 1:**
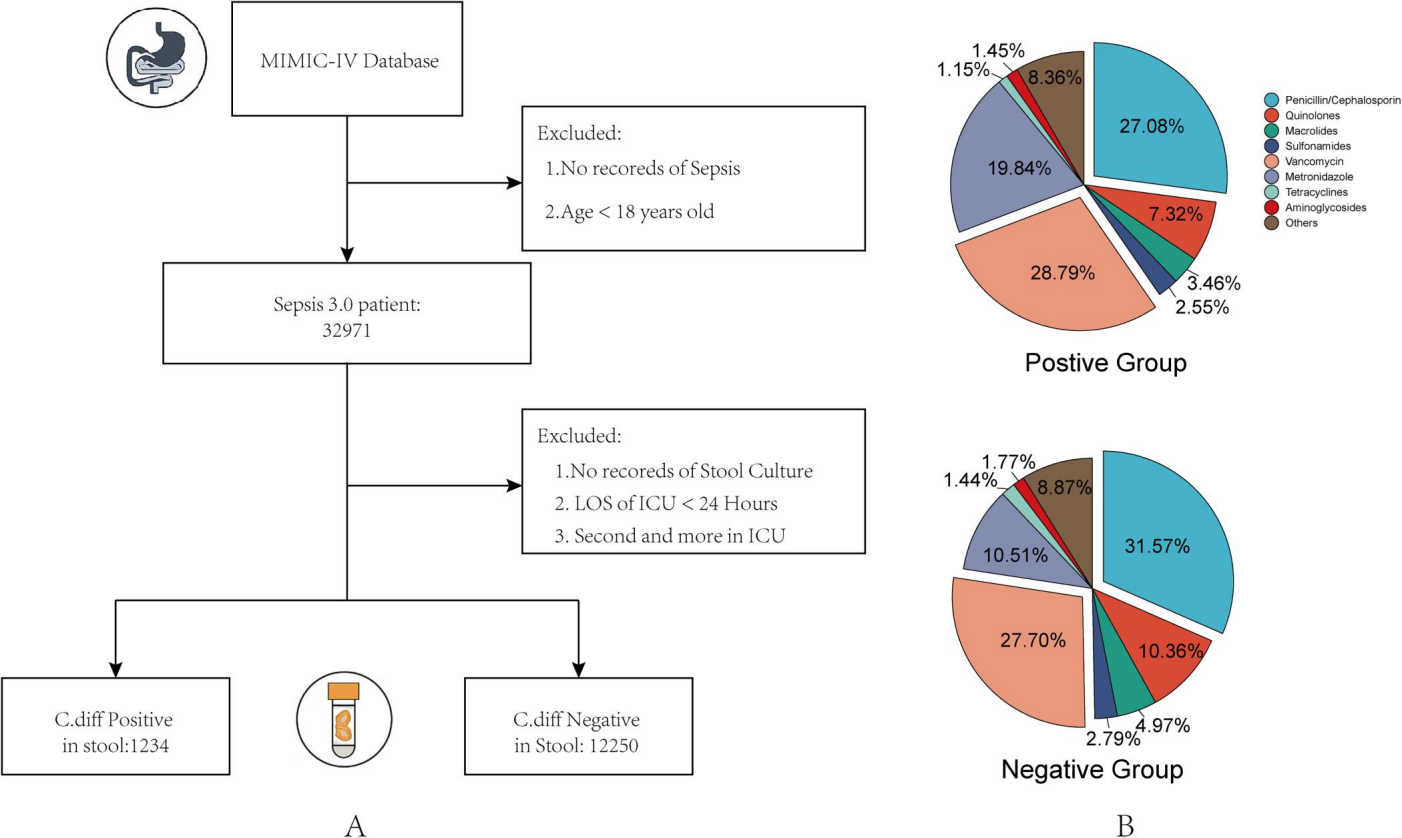
The flow of the study. (A) Patient enrollment flowchart. (B) Distribution of antibiotic usage.

Sepsis 3.0 defines sepsis as organ dysfunction caused by infection, characterized by an elevated Sequential Organ Failure Assessment (SOFA) score of ≥ 2, and suspected infection [1]. Suspected infection is confirmed by blood culture, and preemptive antibiotic therapy is initiated. Further details regarding on the specific code and paper plan can be found on Git‐hub [24]. For patients with multiple ICU hospitalizations, only the first hospitalization record was considered in our analysis. Patients who met any of the following criteria were excluded from the study: age less than 18 years, ICU stay duration < 24 h, and absence of stool pathogen examination records. The flowchart of the patient inclusion process is shown in Figure 1.

### 2.3 Data Extraction and Definitions

Demographic characteristics, vital signs, comorbidities, treatments during ICU stay, and laboratory measurements were extracted using Navicat Premium software (version 15.0.28) and Structured Query Language. Exposure (laboratory‐detected *C. difficile* stool‐test positivity). The primary exposure was any laboratory‐detected *C. difficile*‐positive stool test during the index hospitalization. In MIMIC‐IV, stool microbiology was identified from the microbiology events table using stool specimen descriptors (e.g., spec_type_desc) consistent with stool specimens, *C. difficile* assay names in test_name, and organism/result fields (org_name) indicating *C. difficile* or a positive result. To address possible misclassification of colonization as infection, positive tests were further classified by assay label: toxin‐positive tests included *Clostridium difficile* toxin A and B test, *Clostridium difficile* toxin assay, and *C. difficile* toxin antigen assay; PCR‐positive tests included *C. difficile* PCR. PCR‐only positivity was defined as a positive PCR result without any toxin‐positive *C. difficile* test during the same index hospitalization. We also identified CDI diagnosis codes (ICD‐9 00845; ICD‐10 A047, A0471, A0472) and a treatment‐support proxy defined as oral/enteral vancomycin or fidaxomicin from 24 h before to 7 days after the first positive stool test. Structured diarrhea documentation and endoscopic findings were not sufficiently available to adjudicate clinical CDI. Antibiotic exposure was extracted from MIMIC‐IV derived antibiotics and classified as fluoroquinolone, cephalosporin, clindamycin, carbapenem, piperacillin‐tazobactam, vancomycin, or metronidazole. Vancomycin was further separated by route as intravenous versus oral/enteral when route information was available. To reduce reverse causality from CDI‐directed therapy, early antibiotic exposure for confounding adjustment was defined from 24 h before to 48 h after suspected infection/sepsis recognition, whereas oral/enteral vancomycin, fidaxomicin, and metronidazole around the first positive *C. difficile* test were interpreted as treatment‐support proxies rather than upstream risk exposures. A comprehensive list of assay names, synonyms, and query strings used to flag eligible records is provided in Supporting Information S1: Table S1. To address exposure timing, we generated additional indicators for *C. difficile* positivity during the ICU stay, first positivity within 48 h after suspected infection/sepsis recognition, and 48‐h landmark eligibility. Among 1234 *C. difficile*‐positive stays in the current index‐hospitalization extraction, 707 had the first positive test during the ICU stay, and 567 had the first positive test within 48 h after suspected infection; the median interval from suspected infection to first positive test was 61.4 h (IQR, 11.0–171.6). Primary endpoints were (i) in‐hospital mortality and (ii) 30‐ and 90‐day survival after hospital discharge. Day 0 was defined as the calendar day of discharge from the index hospitalization.

Exposure‐ascertainment audit. The extraction logic was internally reproduced across 56,372 source rows and 16,677 linked assay‐row‐by‐stay records; 1345 positive rows mapped to 1234 positive stays. The audit identified 24 exact‐duplicate groups (48 rows) and 33 same‐stay‐time‐test groups (66 rows); these were collapsed before stay‐level classification, and each stay contributed one binary exposure with the first positive timestamp used in timing analyses.

### 2.4 Association and Incremental‐Prediction Analyses

All *C. difficile*‐specific analyses added during revision were post hoc. Conventional Cox models adjusted sequentially for age, sex, ethnicity, SOFA score, Charlson comorbidity index, acute kidney injury, mechanical ventilation, vasoactive therapy, and eight early‐antibiotic indicators (fluoroquinolone, cephalosporin, clindamycin, carbapenem, piperacillin‐tazobactam, intravenous vancomycin, oral/enteral vancomycin, and metronidazole); ICU length of stay was evaluated only in a sensitivity analysis. Stabilized inverse probability weighting used the same covariates, and a standardized mean difference below 0.10 indicated acceptable balance. For incremental prediction, a baseline logistic model containing age, sex, ethnicity, SOFA, minimum GCS, Charlson index, acute kidney injury, ventilation, and vasoactive therapy was compared with an otherwise identical model adding *C. difficile* positivity. Discrimination, Brier score, and calibration were estimated using 1000 subject‐clustered bootstrap samples with paired out‐of‐bag predictions. Continuous net reclassification improvement was exploratory; category‐based NRI was not calculated because no prespecified clinical risk thresholds were available.

### 2.5 Exploratory Positive‐Subgroup Prediction Models

The archived Cox/Lasso and machine‐learning analyses were restricted to *C. difficile*‐positive stays. Because positivity did not vary within these models, they addressed only risk stratification after a positive test and could not evaluate whether positivity improved prediction beyond conventional severity measures.

Eight machine‐learning algorithms were explored for in‐hospital and 30‐day mortality within *C. difficile*‐positive stays. The archived workflow used a 7:3 stay‐level split followed by model comparison and tuning. Because a fully independent external test cohort and confirmed patient‐level separation were not available, the reported performance is considered exploratory and potentially optimistic. SHAP summaries were used descriptively and not as evidence of causality or treatment targets.

### 2.6 Statistical Methods

Missing values in the archived analytic dataset were imputed by chained equations with predictive mean matching (*m* = 5, maximum iterations = 50, seed = 2023); archived downstream analyses used the first completed dataset. The reconstructed matrices used for the post hoc association and incremental‐prediction analyses had no residual missing model covariates. Baseline variables were compared using independent‐samples *t*‐tests or Mann–Whitney *U* tests as appropriate. Survival probabilities were estimated by Kaplan–Meier methods and compared using log‐rank tests. Cox and stabilized‐weighted models used the specifications described above. All *C. difficile*‐specific exposure‐audit, association, timing, assay, weighting, incremental‐prediction, and positive‐subgroup machine‐learning analyses were post hoc; the continuous‐NRI and machine‐learning analyses were additionally exploratory. Tests were two‐sided with alpha = 0.05.

## 3 Results

### 3.1 Patients Characteristics

Table 1 summarizes the full cohort of 13,484 index hospitalizations, including 1234 *C. difficile*‐positive stays and 12,250 comparator/no‐positive stays. The comparator included stays without a linked positive result and was not uniformly assay‐negative. Baseline and treatment differences are descriptive and do not establish an independent effect of laboratory positivity.

**Table 1:** Baseline characteristics of the full index‐hospitalization cohort.

Variables	Total (*n* = 13,484)	Comparator/no‐positive (*n* = 12,250)	*C. difficile* positive (*n* = 1234)	*p* value
Age, median (Q1, Q3)	67.4 (56.46,78.02)	67.38 (56.37, 77.86)	67.96 (57.42, 79.27)	0.057
Weight, median (Q1, Q3)	77.6 (65, 93.7)	77.8 (65.2, 94)	75.9 (64.21, 91.85)	0.012
Sex, *n* (%)				0.337
Female	5983 (44)	5419 (44)	564 (46)	
Male	7501 (56)	6831 (56)	670 (54)	
Ethnicity, *n* (%)				0.009
White	9180 (68)	8355 (68)	825 (67)	
Black	1599 (12)	1421 (12)	178 (14)	
Asian	556 (4)	518 (4)	38 (3)	
Other race	2149 (16)	1956 (16)	193 (16)	
SOFA, median (Q1, Q3)	6 (4, 9)	6 (4, 9)	6 (4, 9)	0.343
GCS, median (Q1, Q3)	15 (13, 15)	15 (13, 15)	15 (13, 15)	0.679
Charlson comorbidity index, median (Q1, Q3)	6 (4, 8)	6 (4, 8)	6 (4, 8)	< 0.001
WBC, median (Q1, Q3)	9.8 (6.5, 14)	9.7 (6.4, 13.9)	10.6 (6.9, 15.6)	< 0.001
BUN, median (Q1, Q3)	23 (15, 39)	23 (14, 39)	24.5 (15, 42)	0.049
Creatinine, median (Q1, Q3)	1.1 (0.7, 1.8)	1.1 (0.7, 1.8)	1.1 (0.7, 2.1)	0.065
Heart rate, median (Q1, Q3)	73 (63, 85)	73 (63, 84)	75 (64, 87)	< 0.001
SBP, median (Q1, Q3)	87 (78, 97)	87 (78, 97)	85 (77, 94)	< 0.001
DBP, median (Q1,Q3)	44 (37, 50)	44 (37, 50)	43 (37, 50)	0.209
Resp rate, median (Q1, Q3)	13 (10, 15)	13 (10, 15)	13 (10.12, 16)	0.154
Temperature, median (Q1, Q3)	36.86 (36.59, 37.21)	36.86 (36.59, 37.21)	36.87 (36.6, 37.21)	0.44
Spo2, median (Q1, Q3)	92 (90, 95)	92 (90, 95)	92.5 (89.25, 95)	0.698
Glucose, median (Q1, Q3)	133.53 (110.5, 167.25)	133.5 (110.75, 167.2)	134.31 (108.25, 167.49)	0.362
Urine output, median (Q1, Q3)	1289.5 (685, 2105)	1300 (700, 2129.75)	1140 (520.75, 1970)	< 0.001
AKI, *n* (%)				0.002
None	6146 (46)	5642 (46)	504 (41)	
I	3696 (27)	3348 (27)	348 (28)	
II	1295 (10)	1164 (10)	131 (11)	
III	2347 (17)	2096 (17)	251 (20)	
Ventilation, *n* (%)				0.071
No use	5332 (40)	4814 (39)	518 (42)	
Use	8152 (60)	7436 (61)	716 (58)	
Vasoactive, *n* (%)				0.012
No use	6531 (48)	5976 (49)	555 (45)	
Use	6953 (52)	6274 (51)	679 (55)	
Penicillin/cephalosporin, *n* (%)				0.006
No use	2467 (18)	2205 (18)	262 (21)	
Use	11,017 (82)	10,045 (82)	972 (79)	
Quinolones, *n* (%)				< 0.001
No use	9927 (74)	8947 (73)	980 (79)	
Use	3557 (26)	3303 (27)	254 (21)	
Macrolides, *n* (%)				0.001
No use	11,779 (87)	10,664 (87)	1115 (90)	
Use	1705 (13)	1586 (13)	119 (10)	
Sulfonamides, *n* (%)				0.483
No use	12,505 (93)	11,354 (93)	1151 (93)	
Use	979 (7)	896 (7)	83 (7)	
Vancomycin, *n* (%)				< 0.001
No use	3631 (27)	3439 (28)	192 (16)	
Use	9853 (73)	8811 (72)	1042 (84)	
Metronidazole, *n* (%)				< 0.001
No use	9412 (70)	8898 (73)	514 (42)	
Use	4072 (30)	3352 (27)	720 (58)	
Tetracyclines, *n* (%)				0.032
No use	12,879 (96)	11,685 (95)	1194 (97)	
Use	605 (4)	565 (5)	40 (3)	
Aminoglycosides, *n* (%)				0.459
No use	12,973 (96)	11,791 (96)	1182 (96)	
Use	511 (4)	459 (4)	52 (4)	
Other antibiotics, *n* (%)				0.453
No use	10,360 (77)	9423 (77)	937 (76)	
Use	3124 (23)	2827 (23)	297 (24)	
In‐hospital outcome, *n* (%)				< 0.001
Alive	11,156 (83)	10,184 (83)	972 (79)	
Died	2328 (17)	2066 (17)	262 (21)	
90‐day outcome, *n* (%)				< 0.001
Alive	7744 (57)	7123 (58)	621 (50)	
Died	5740 (43)	5127 (42)	613 (50)	

### 3.2 Antibiotic Usage

Antibiotic exposure differed between groups. Compared with comparator/no‐positive stays, *C. difficile*‐positive stays had lower use of penicillin/cephalosporin (79% vs. 82%; *p* = 0.006), quinolones (21% vs. 27%; *p* < 0.001), macrolides (10% vs. 13%; *p* = 0.001), and tetracyclines (3% vs. 5%; *p* = 0.032), and higher use of vancomycin (84% vs. 72%; *p* < 0.001) and metronidazole (58% vs. 27%; *p* < 0.001). These crude differences may reflect indication, illness severity, and testing opportunity.

### 3.3 Survival by *C*. *difficile* Stool‐Test Status

**Figure 2:**
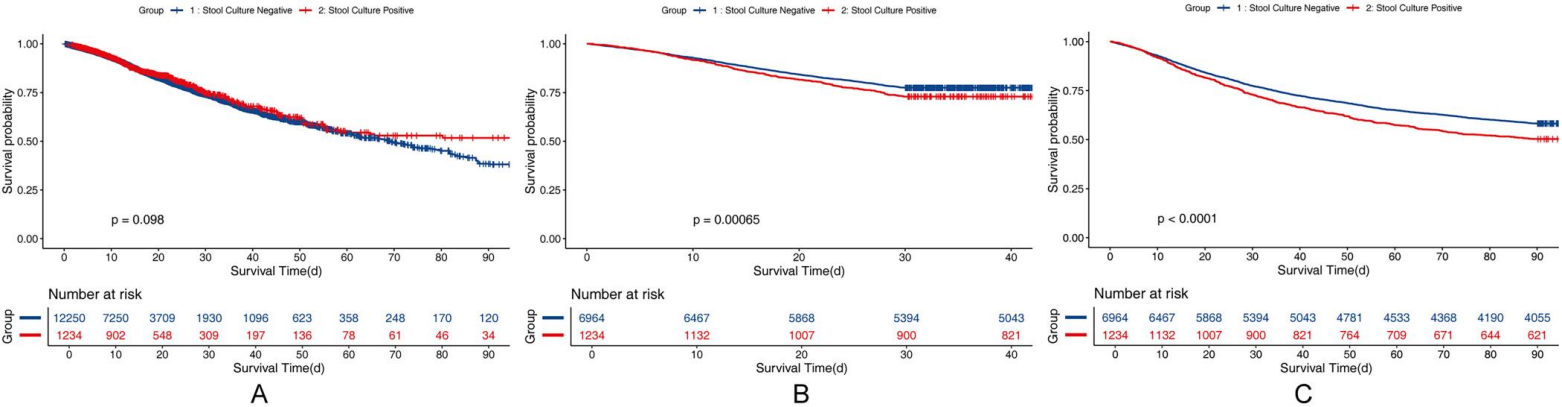
Descriptive archived Kaplan–Meier curves by *C. difficile* stool‐test status. (A) In‐hospital survival; (B) 30‐day survival; (C) 90‐day survival.

The Kaplan–Meier curves in Figure 2 illustrate survival probabilities among sepsis patients stratified by laboratory‐detected *C. difficile* stool‐test status. In‐hospital survival did not differ significantly between the *C. difficile*‐negative and *C. difficile*‐positive groups (Figure 2A). However, 30‐day and 90‐day survival curves separated after discharge, with lower survival among patients with *C. difficile* stool‐test positivity (Figure 2B,C). These findings suggest that *C. difficile*‐related stool‐test signals may provide prognostic information beyond the acute hospitalization, although the observational design and variability in testing timing require cautious interpretation.

### 3.4 Cox Regression

**Figure 3:**
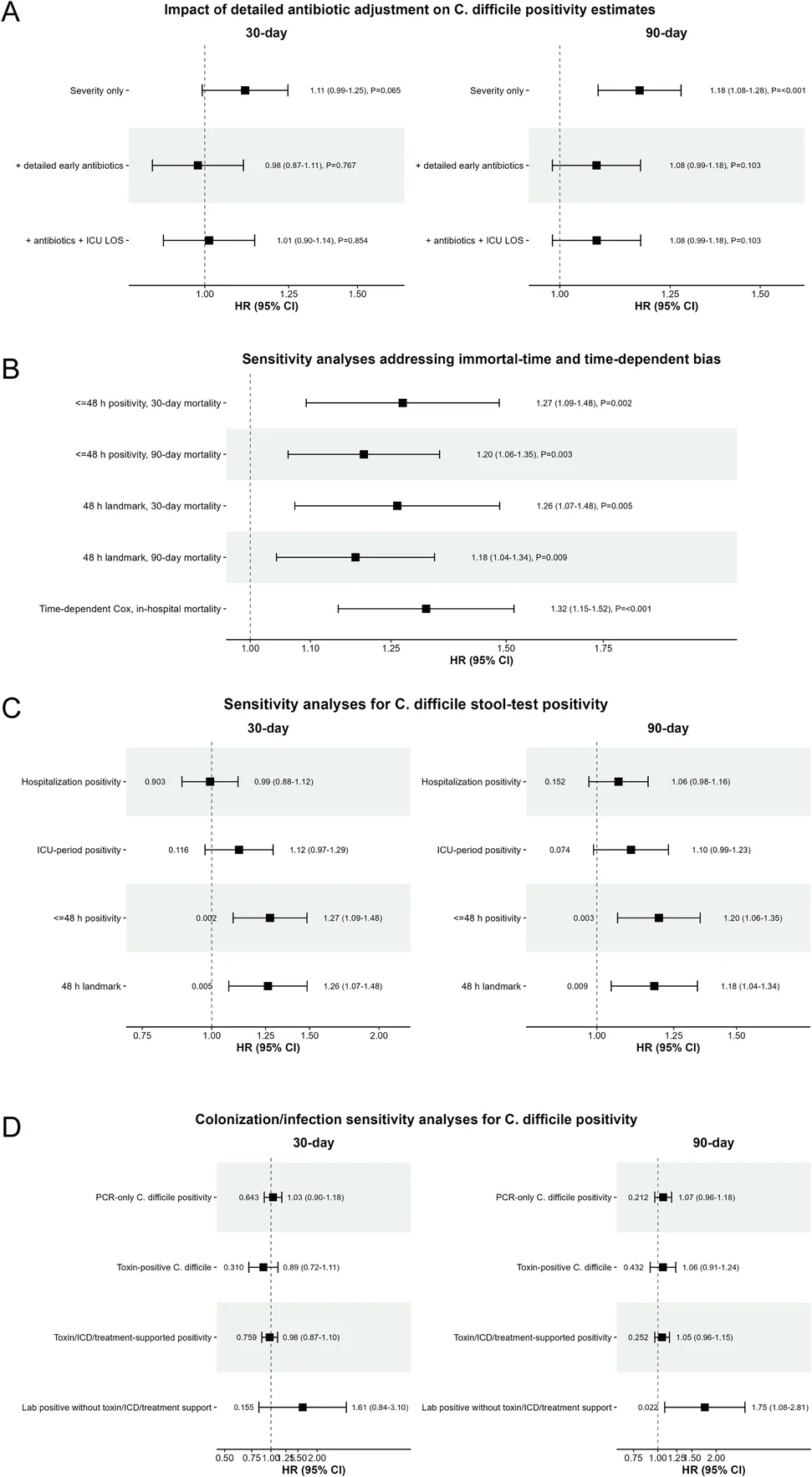
Archived sensitivity‐analysis display. (A) Detailed antibiotic adjustment; (B, C) timing analyses from archived model stages; (D) assay/support‐defined analyses.

The association was substantially attenuated after detailed early‐antibiotic adjustment (Figure 3A). In a clinical‐severity model, index‐hospitalization positivity was borderline for 30‐day mortality (HR, 1.11; 95% CI, 0.99–1.25; *p* = 0.065) and associated with 90‐day mortality (HR, 1.18; 95% CI, 1.08–1.28; *p* < 0.001). After adding eight early‐antibiotic indicators, the estimates were null for 30‐day mortality (HR, 0.98; 95% CI, 0.87–1.11; *p* = 0.767) and 90‐day mortality (HR, 1.08; 95% CI, 0.99–1.18; *p* = 0.103). Additional adjustment for ICU length of stay produced similar results. Fully antibiotic‐adjusted early‐positivity estimates were also nonsignificant at 30 days (HR, 1.15; 95% CI, 0.97–1.37; *p* = 0.102) and 90 days (HR, 1.12; 95% CI, 0.98–1.28; *p* = 0.098).

In the fully adjusted 48‐h landmark analysis, early positivity was not associated with 30‐day mortality (HR, 1.13; 95% CI, 0.94–1.36; *p* = 0.181) or 90‐day mortality (HR, 1.11; 95% CI, 0.96–1.27; *p* = 0.164). The time‐dependent in‐hospital Cox model remained associated with mortality (HR, 1.28; 95% CI, 1.10–1.48; *p* < 0.001), but this model used a different time origin. PCR‐only positivity was null at 30 days (HR, 1.03; 95% CI, 0.90–1.18; *p* = 0.643) and 90 days (HR, 1.07; 95% CI, 0.96–1.18; *p* = 0.212); toxin‐positive status was likewise null at 30 days (HR, 0.89; 95% CI, 0.72–1.11; *p* = 0.310) and 90 days (HR, 1.06; 95% CI, 0.91–1.24; *p* = 0.432). The archived timing panels in Figure 3 include earlier model stages; the fully detailed‐antibiotic‐adjusted estimates reported here and in Supporting Information S2: Table S2 are used for interpretation.

### 3.5 Sensitivity and Incremental‐Prediction Summary

Stabilized weighting improved measured balance (mean absolute SMD, 0.026; maximum absolute SMD, 0.064; no covariate above 0.10). The weighted 30‐day association was null (HR, 0.99; 95% CI, 0.86–1.13; *p* = 0.832), whereas the 90‐day estimate was modest (HR, 1.13; 95% CI, 1.03–1.24; *p* = 0.010). At 30 days, AUC was 0.7138 versus 0.7140 (Delta AUC, 0.00017; 95% CI, −0.00156 to 0.00200); at 90 days, AUC was 0.7053 versus 0.7052 (Delta AUC, −0.00006; 95% CI, −0.00114 to 0.00111). Brier‐score changes were negligible, and calibration was essentially unchanged, indicating no meaningful incremental predictive information (Supporting Information S2: Table S2).

### 3.6 Exploratory Positive‐Subgroup Prediction Models

**Figure 4:**
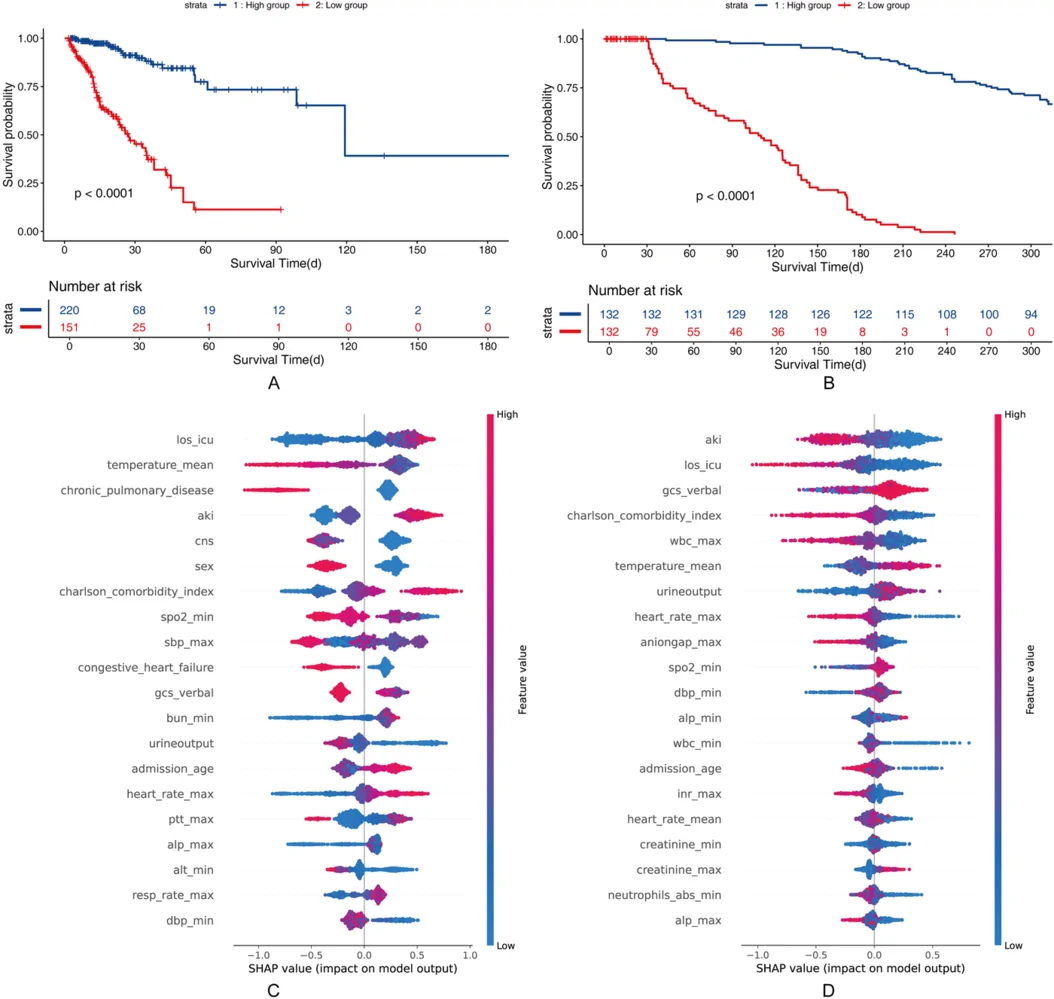
Exploratory within‐positive‐stay risk stratification. (A, B) Archived high‐ versus low‐risk Kaplan–Meier curves; (C, D) descriptive SHAP feature‐attribution summaries for in‐hospital and 30‐day mortality.

The archived positive‐subgroup Cox/Lasso model yielded test‐set C‐indices of 0.81 for in‐hospital mortality and 0.68 for 30‐day mortality. These estimates describe risk stratification only among *C. difficile*‐positive stays and do not show that positivity improves prediction beyond severity measures. Figure 4A,B shows descriptive separation of archived high‐ and low‐risk groups.

Eight algorithms were compared in the archived positive‐subgroup workflow, with optimized test‐set AUCs of 0.94 for in‐hospital and 0.78 for 30‐day mortality. Because model comparison, tuning, and evaluation occurred within one archived workflow, these values are reported as exploratory rather than evidence of robustness, superiority, clinical utility, or external validation. The SHAP panels in Figure 4C,D are descriptive feature‐attribution summaries and should not be interpreted causally or as treatment targets.

## 4 Discussion

This study evaluated the association between stool‐test positivity and in‐hospital, 30‐day, and 90‐day mortality in patients with sepsis using a large contemporary critical‐care cohort. The results showed that patients with positive stool‐test results, predominantly laboratory‐detected *C. difficile*, had increased mortality rates across these time horizons, but the association was substantially attenuated after detailed antibiotic adjustment and was inconsistent across timing‐, assay‐, and weighting‐based analyses. We also developed a machine‐learning model to predict in‐hospital and 30‐day mortality in sepsis patients with *C. difficile* stool‐test positivity. Light GBM was the best‐performing algorithm, achieving an AUC of 0.94 for in‐hospital mortality and 0.78 for 30‐day mortality after hyperparameter optimization. SHAP analysis identified ICU length of stay, Charlson comorbidity index, acute kidney injury, temperature, neutrophil count, and urine output as important predictors. Importantly, these variables should be interpreted as risk‐stratification signals rather than direct therapeutic targets: the Charlson comorbidity index reflects baseline host vulnerability, and ICU length of stay is largely a downstream marker of illness severity, complications, and recovery trajectory. Their clinical value is to identify septic patients with *C. difficile* stool‐test positivity who may benefit from earlier bedside review, closer monitoring, microbiology‐guided antimicrobial stewardship, infection‐control measures, organ‐support optimization, and structured post‐ICU follow‐up.

**Figure 5:**
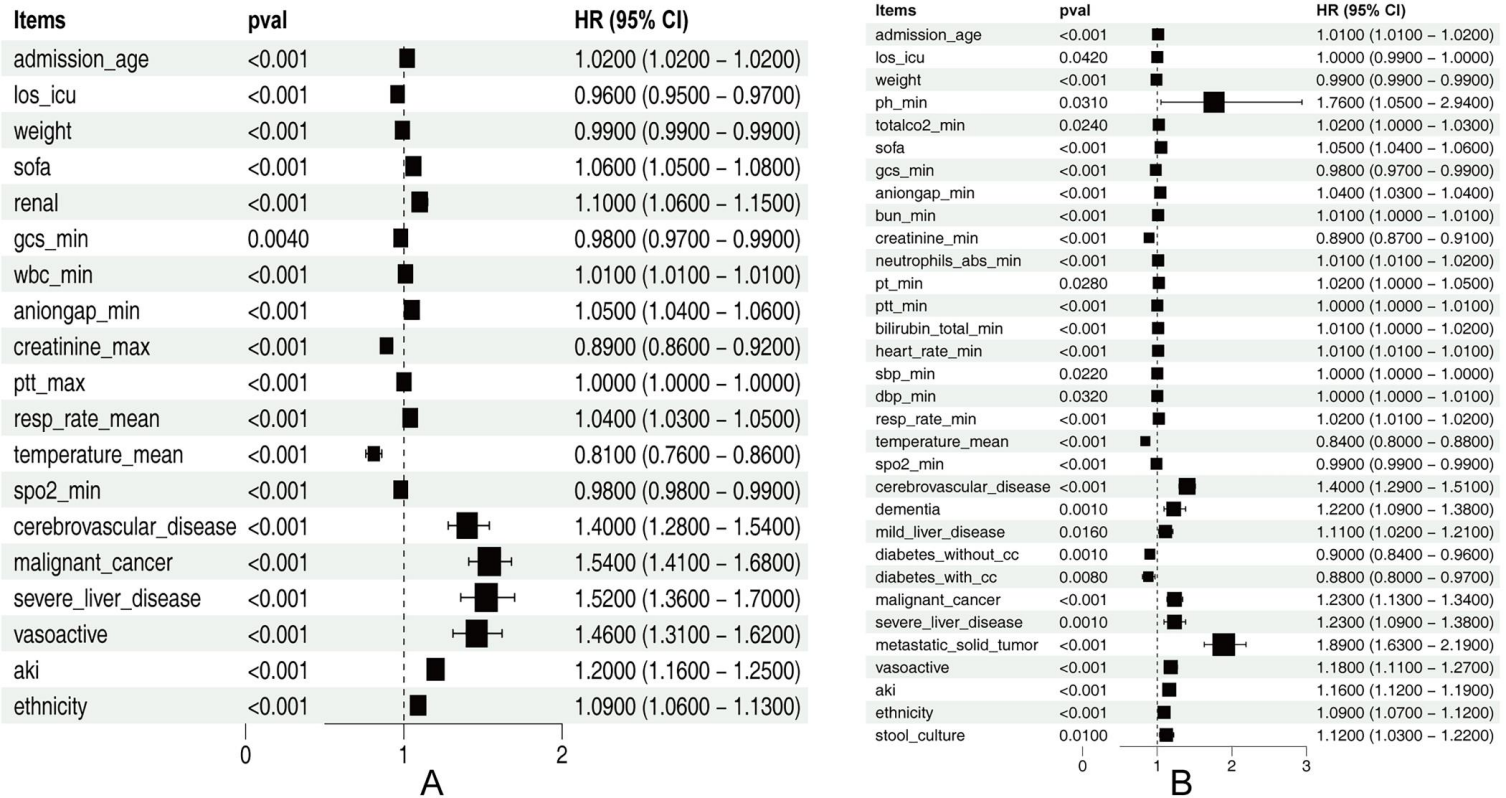
Multivariable Cox regression forest plot. (A) 30‐day survival in sepsis patients; (B) 90‐day survival in sepsis patients.

There have been no reports on stool pathogens in sepsis patients in the past. In this large critical‑care cohort, our model's C‑index of 0.68 for 30‑day mortality—though modest—compares favorably to established sepsis tools. The SOFA score, widely used for risk stratification, achieved a discrimination of 0.62 in the same dataset, while the GCS alone performed less robustly (C‑index 0.55). Notably, our model integrates both conventional physiologic parameters and novel laboratory biomarkers not captured by SOFA or GCS, which likely underlies its improved prognostic performance. Our forest plots (Figure 5A,B) quantify the independent contribution of routinely available ICU variables to post‐discharge mortality in sepsis. The direction and magnitude of these associations mirror the biological plausibility we expect at the bedside, thereby reinforcing model validity. Meanwhile, the SHAP value also demonstrates the important factors that the model focuses on.

The clinical application of the model therefore lies in translating risk signals into modifiable care processes. For patients with a high Charlson comorbidity index or a prolonged ICU trajectory, the actionable response is not to modify the index itself, but to intensify *C. difficile*‐focused sepsis management: prompt confirmation of *C. difficile* when clinically suspected, contact precautions, review and de‐escalation of unnecessary broad‐spectrum antibiotics, appropriate CDI therapy when infection is suspected or confirmed, optimization of hemodynamics, oxygenation, renal support, and fluid balance, prevention of device‐related and other nosocomial complications, and planned transition‐of‐care surveillance after ICU discharge. In this framework, *C. difficile* remains clinically central rather than being displaced by other predictors. Stool‐test positivity identifies a gut‐related infectious and dysbiosis signal that may compound sepsis recovery, whereas comorbidity burden and ICU length of stay identify the vulnerable host context in which this signal is most consequential. Future work should test whether embedding real‐time *C. difficile* stool‐test results into sepsis bundles can reduce avoidable ICU complications and improve 30‐ and 90‐day survival.

More recently, mechanistic work has shown that shedding of ACE2 in sepsis contributes to loss of microbial‐derived metabolites—including 5‑methoxytryptophan—thereby disrupting gut barrier integrity and exacerbating systemic inflammation in models of gut leak syndrome [25]. This underscores a biologically plausible pathway whereby sepsis can both result from and drive dysbiosis. Another recent MR investigation demonstrated that the presence of *Coprococcus1, Coprococcus2*, and *Anaerostipes* is associated with decreased sepsis incidence and reduced 28‐day mortality among ICU‐level patients, whereas *Ruminococcus torques* subgroups and *Sellimonas* increased the risk [2]. Taken together, these emerging data strengthen the potential causal role of specific microbiota taxa in both susceptibility to and severity of sepsis.

Laboratory‐detected *C. difficile* stool‐test positivity was associated with worse 30‐day and 90‐day outcomes among patients with sepsis. Prior studies of the intestinal microbiome in sepsis have shown that gut microbial diversity and richness are reduced early after admission, indicating rapid disruption of the intestinal microbial ecosystem rather than expansion of pathogenic bacteria alone [26]. Intestinal amino acid metabolism is also altered in patients with sepsis compared with healthy controls, reflecting metabolic imbalance accompanying critical illness. In our cohort, *C. difficile* was the predominant organism among positive stool records, whereas other enteric organisms were uncommon and heterogeneous; therefore, we did not perform formal organism‐specific comparative modeling. *C. difficile* is a gram‐positive, spore‐forming, anaerobic bacillus that is widely distributed in the intestines of humans and animals and in the environment. Transmission occurs through the fecal‐oral route, and major risk factors for *C. difficile* colonization or infection include antibiotic exposure, older age, hospitalization, and residence in a nursing home [27].

The antibiotic findings require route‐ and timing‐specific interpretation. Antibiotic exposure is a major risk factor for CDI and may also mark severe infection, prolonged hospitalization, and greater opportunity for stool testing. In the detailed antibiotic‐adjusted analyses, adding early fluoroquinolone, cephalosporin, clindamycin, carbapenem, piperacillin‐tazobactam, intravenous vancomycin, oral/enteral vancomycin, and metronidazole variables attenuated the conventional 30‐day and 90‐day Cox estimates for *C. difficile* stool‐test positivity. This suggests that part of the unadjusted or less‐adjusted prognostic signal may reflect antibiotic pressure, illness severity, and treatment indication rather than an independent biologic effect of *C. difficile* alone. Intravenous vancomycin is commonly used in critically ill sepsis patients for suspected or confirmed resistant Gram‐positive infections, including methicillin‐resistant Staphylococcus aureus, whereas oral or enteral vancomycin is used for suspected or confirmed CDI. Metronidazole may also represent CDI‐directed treatment rather than an upstream risk exposure. Therefore, observed associations involving vancomycin or metronidazole should not be interpreted as evidence that these drugs caused *C. difficile* positivity. Clinically, these findings support microbiology‐guided antimicrobial stewardship: initiate empiric therapy when indicated for sepsis, but reassess and de‐escalate within 48–72 h according to culture results, local antibiograms, and the likelihood of CDI.

*C. difficile* stool‐test positivity was associated with decreased survival in the primary analyses, but this association should be interpreted cautiously. A positive laboratory test may represent toxin‐mediated CDI, colonization, or testing/treatment selection in a high‐risk ICU population. To explore this concern, we separated toxin‐positive results from PCR‐only positivity and incorporated structured CDI‐support evidence, including ICD‐coded CDI and oral/enteral vancomycin or fidaxomicin near the first positive test. Most positive stays had at least one supportive feature, but toxin‐positive and PCR‐only subgroups were not independently associated with mortality after adjustment for clinical severity and antibiotic exposure. These findings support the prognostic relevance of *C. difficile*‐related stool‐test signals in sepsis but argue against interpreting all positive tests as active infection. The retrospective design and absence of longitudinal pre‐illness microbiome sampling preclude determination of whether dysbiosis preceded sepsis or resulted from critical illness, antibiotic exposure, and prolonged hospitalization. Further prospective studies with standardized CDI diagnostic criteria are needed.

Although in‑hospital mortality did not differ between stool culture‐positive and ‐negative patients, our 30‑day and 90‑day mortality analyses—derived from linkage to national death registries—capture both in‑hospital and post‑discharge events. Early mortality in sepsis is driven predominantly by acute‑phase factors such as hemodynamic instability, the need for vasopressor and ventilatory support, and prompt administration of appropriate antimicrobials; when these interventions are equally optimized, in‑hospital survival curves tend to converge. By contrast, as patients move beyond the ICU, subacute processes—including persistent immune dysregulation, ongoing microbial translocation, progressive organ dysfunction, secondary nosocomial infections, and failure of gut‑barrier integrity—become the key determinants of outcome [26]. These late‑phase complications often differ in prevalence and severity between culture‑positive and culture‑negative cohorts, thus explaining why statistically significant separation in mortality only emerges at 30 and 90 days despite comparable in‑hospital survival.

The incremental clinical value of *C. difficile* stool‐test positivity lies in the disease‐specific and actionable information that it contributes beyond conventional severity measures. Scores based on physiological abnormalities, organ dysfunction, and comorbidity describe the overall severity of sepsis but do not identify intestinal infection, antibiotic‐associated disruption of the gut environment, or the potential need for CDI‐specific management. Thus, among patients with otherwise similar severity profiles, positivity may identify an additional infection‐related risk pathway involving possible toxin‐mediated intestinal injury, gastrointestinal fluid loss, continued antibiotic pressure, and impaired recovery of the intestinal ecosystem. Recognition of this pathway can refine clinical prognostic assessment and alter management priorities, including adjudication of active CDI, monitoring of gastrointestinal and volume status, antimicrobial review and de‐escalation, timely targeted treatment, contact precautions, and post‐discharge surveillance. *C. difficile* positivity should therefore be regarded as a clinically meaningful complement to established severity measures rather than as a replacement for them. Its interpretation nevertheless requires integration with diarrhea and stool characteristics, toxin evidence, recent antibiotic exposure, and alternative causes of gastrointestinal symptoms.

A primary strength of this study was the size and clinical detail of MIMIC‐IV. Several limitations remain. First, the exposure algorithm underwent an internal reproducibility audit but not formal clinical validation; no separate clinical‐order linkage, manual chart review, diarrhea adjudication, or external gold standard was available. Exact and same‐stay‐time‐test duplicates were collapsed before stay‐level classification, but residual misclassification is possible, and the comparator was not uniformly assay‐negative. Second, testing time varied, leaving residual time‐dependent and immortal‐time bias despite early, landmark, and time‐dependent analyses. Third, detailed antibiotic adjustment reduced but did not eliminate confounding by indication. Fourth, archived preprocessing used multiple imputation, but downstream models used only the first completed dataset rather than Rubin‐rule pooling. Fifth, the positive‐subgroup machine‐learning analyses were internally evaluated without external validation and may be optimistic.

## 5 Conclusion

While laboratory‐detected *C. difficile* stool‐test positivity correlates with poorer unadjusted 30‐day and 90‐day survival among septic patients, rigorous antibiotic adjustment and sensitivity analyses demonstrate that this association is substantially attenuated and inconsistent. Given that our exposure definition does not establish clinically confirmed CDI, laboratory positivity may serve primarily as a marker of a complex clinical phenotype. Ultimately, evidence for its independent prognostic utility remains limited, and its incremental predictive value beyond conventional severity measures is minimal. Prospective investigations incorporating standardized CDI diagnostic criteria, specific antibiotic exposure windows, and longitudinal microbiome tracking are essential before translating these findings into clinical practice.

## Author Contributions

**Jiyun Hu:** writing – review and editing, writing – original draft. **Fuxing Deng:** methodology. **Tingyue Hu:** validation. **Binbin Meng:** data curation. **Songyun Deng:** visualization, validation. **Lina Zhang:** funding acquisition. **Wenchao Li:** writing – review and editing, writing – original draft.

## Conflicts of Interest

The authors declare no conflicts of interest.

## Transparency Statement

The lead author (Jiyun Hu) and corresponding author (Wenchao Li) affirm that this manuscript is an honest, accurate, and transparent account of the study being reported, that no important aspects of the study have been omitted, and that any discrepancies from the study as planned (and, if relevant, registered) have been explained.

## Supporting information


Supporting File 1

Supporting File 2

## Data Availability

The data are open. The data that support the findings of this study are available in MIMIC IV database at https://mimic.mit.edu. These data were derived from the following resources available in the public domain: MIMIC IV database, https://mimic.mit.edu.
