# Supplementary material for: Laboratory‐Detected Clostridioides difficile Stool‐Test Positivity and Mortality in Sepsis: A Retrospective Cohort Study
Source: Health Sci Rep. 2026 Sep 7;9(9):e73206. doi: 10.1002/hsr2.73206 (PMC13550520; doi:10.1002/hsr2.73206)
Supplement: Supplementary file 1 [file HSR2-9-e73206-s002.docx]

**Supplementary Table S2. Post hoc association and incremental-prediction analyses**

| **Analysis** | **Outcome** | **Base/comparator** | **Positive/extended** | **Estimate or change** |
| --- | --- | --- | --- | --- |
| Detailed-antibiotic Cox | 30-day / 90-day mortality | No positivity (reference) | HR 0.98 (0.87-1.11) / HR 1.08 (0.99-1.18) | P=0.767 / P=0.103; both CIs include 1 |
| Stabilized IPW | 30-day / 90-day mortality | No positivity (reference) | HR 0.99 (0.86-1.13) / HR 1.13 (1.03-1.24) | P=0.832 / P=0.010; inconsistent by horizon |
| Nested incremental prediction | 30-day / 90-day mortality | AUC 0.7138 / 0.7053 | AUC 0.7140 / 0.7052 | Delta AUC 0.00017 (-0.00156 to 0.00200) / -0.00006 (-0.00114 to 0.00111) |

Abbreviations: AUC, area under the receiver operating characteristic curve; CI, confidence interval; HR, hazard ratio; IPW, inverse probability weighting. Paired values are presented as 30-day / 90-day outcomes.
